# Warning coloration can be disruptive: aposematic marginal wing patterning in the wood tiger moth

**DOI:** 10.1002/ece3.1736

**Published:** 2015-10-12

**Authors:** Atsushi Honma, Johanna Mappes, Janne K. Valkonen

**Affiliations:** ^1^ University of JyväskyläDepartment of Biological and Environmental ScienceCentre of Excellence in Biological InteractionsP.O. Box 35Jyväskylä40014Finland; ^2^ Department of Ecosystem StudiesSchool of Environmental ScienceThe University of Shiga Prefecture2500 Hassaka‐choHikone CityShiga522‐8533Japan

**Keywords:** Aposematism, camouflage, crypsis, defense, disruptive coloration, predation

## Abstract

Warning (aposematic) and cryptic colorations appear to be mutually incompatible because the primary function of the former is to increase detectability, whereas the function of the latter is to decrease it. Disruptive coloration is a type of crypsis in which the color pattern breaks up the outline of the prey, thus hindering its detection. This delusion can work even when the prey's pattern elements are highly contrasting; thus, it is possible for an animal's coloration to combine both warning and disruptive functions. The coloration of the wood tiger moth (*Parasemia plantaginis*) is such that the moth is conspicuous when it rests on vegetation, but when it feigns death and drops to the grass‐ and litter‐covered ground, it is hard to detect. This death‐feigning behavior therefore immediately switches the function of its coloration from signaling to camouflage. We experimentally tested whether the forewing patterning of wood tiger moths could function as disruptive coloration against certain backgrounds. Using actual forewing patterns of wood tiger moths, we crafted artificial paper moths and placed them on a background image resembling a natural litter and grass background. We manipulated the disruptiveness of the wing pattern so that all (marginal pattern) or none (nonmarginal pattern) of the markings extended to the edge of the wing. Paper moths, each with a hidden palatable food item, were offered to great tits (*Parus major*) in a large aviary where the birds could search for and attack the “moths” according to their detectability. The results showed that prey items with the disruptive marginal pattern were attacked less often than prey without it. However, the disruptive function was apparent only when the prey was brighter than the background. These results suggest that warning coloration and disruptive coloration can work in concert and that the moth, by feigning death, can switch the function of its coloration from warning to disruptive.

## Introduction

Animal coloration has several functions (Endler 1978): It plays important roles in both intraspecific (e.g., mating behavior; Andersson 1994; Houde and Endler 1990; Summers et al. 1999) and interspecific interactions (e.g., predator–prey interactions; Ruxton et al. 2004), and it also may help the animal maintain its physical state (e.g., thermoregulation; Trullas et al. 2007; Lindstedt et al. 2009). Different types of protective coloration are often studied independently despite increasing evidence that they may be interrelated (Stevens 2007). Even two apparently functionally opposite protective coloration types, cryptic (coloration that hinders a predator's ability to detect or recognize the prey) and aposematic (conspicuous coloration that signals the prey's unprofitability), are not necessary mutually exclusive (Wüster et al. 2004). For example, a color pattern may be highly conspicuous and have a warning function at close range, but from a distance, it may be cryptic (Edmunds 1974; Papageorgis 1975; Rothschild 1975; Endler 1978; Järvi et al. 1981; Tullberg et al. 2005; Bohlin et al. 2008). Because aposematic coloration carries the cost of a high detection risk (Gittleman and Harvey 1980; Lindström et al. 1999; Riipi et al. 2001; Summers and Clough 2001; Husak et al. 2006; Mappes et al. 2014), natural selection might favor color patterns that employ both of these defensive tactics.

Disruptive coloration creates the appearance of false edges and also destroys the appearance of the true body edges and outline, which hinders the ability of a predator to detect or recognize an animal by its shape (Thayer 1909; Cott 1940; Stevens and Merilaita 2009b, 2011; Webster et al. 2013). Thus, it can be hypothesized that disruptive coloration is more compatible with warning coloration than other types of crypsis such as background matching (e.g., Järvi et al. 1981) because adjacent markings in disruptive patterns are likely to be more contrasting; therefore, at least some of them may contrast with background colors. However, clear evidence in support of this hypothesis has not yet been presented (Stevens and Merilaita 2009a; Bohlin et al. 2012; Hegna and Mappes 2014).

Disruptive markings can confer a significant survival advantage on prey compared to targets with background‐matching patterns that are not placed disruptively, against both avian (Cuthill et al. 2005, 2006; Merilaita and Lind 2005; Schaefer and Stobbe 2006; Stevens et al. 2006; Stobbe and Schaefer 2008) and human predators (Fraser et al. 2007). One disruptive coloration pattern that has been demonstrated to have a camouflage effect (e.g., Cuthill et al. 2005, 2006; Merilaita and Lind 2005; Fraser et al. 2007) is the “disruptive marginal pattern,” in which the disruptive markings touch the outline of the prey's body (Cott 1940; Stevens and Merilaita 2009b). Disruptive marginal patterns are effective even when some of the pattern elements do not visually match adjacent parts of the background (Cuthill et al. 2005; Schaefer and Stobbe 2006; Stevens et al. 2006; but see Hegna and Mappes 2014). However, some evidence indicates that the survival of individuals with disruptive color patterns characterized by extremely high contrast between adjacent color pattern elements (i.e., “maximum disruptive contrast”; Stevens and Merilaita 2009b) is worse than that of individuals with less contrasting patterns, because the contrast of the pattern elements with background elements is also high (Fraser et al. 2007; Stobbe and Schaefer 2008; Troscianko et al. 2013). Thus, a warning coloration pattern in which adjacent markings are highly contrasting may be more compatible with disruptive than with background‐matching coloration (Järvi et al. 1981).

**Figure 1:**
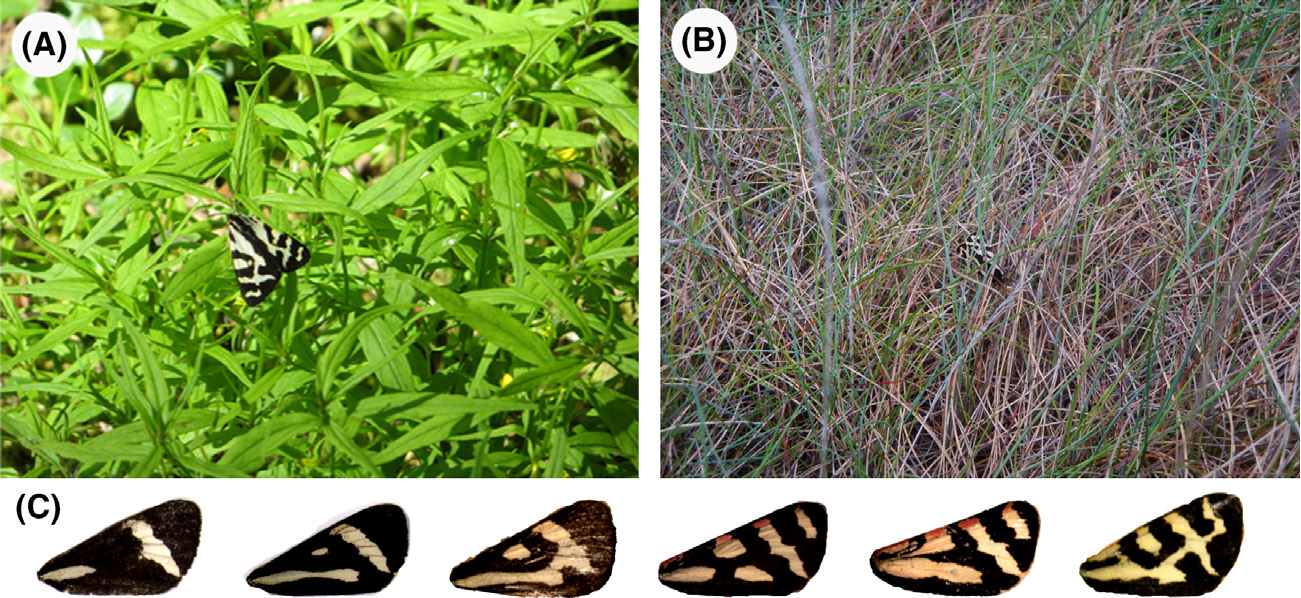
(A) A *Parasemia plantaginis* individual is highly conspicuous resting on a plant. (B) The same individual feigned death and dropped to the ground when the observer approached it, becoming highly cryptic (the moth is in the center of the photo). (C) Geographic variation in forewing patterns (only right forewings are shown). The Regular pattern (rightmost) is common in central and northern Europe, and the Hash pattern (second from the left) is found mainly in North America.

The wood tiger moth (*Parasemia plantaginis*) is an aposematic species with a characteristic forewing color pattern, and its hindwing coloration has been demonstrated to function as a warning signal in both laboratory and field experiments (Lindstedt et al. 2011; Nokelainen et al. 2012; Hegna et al. 2013). Although the appearance of the wood tiger moth varies geographically, some of its color morphs, including one prominent in northern Europe, seem to have the characteristics of disruptive coloration (i.e., high‐contrast markings overlapping the wing edge; Hegna and Mappes 2014). Adult individuals are highly conspicuous on green foliage, their typical resting spot in the wild (Fig. 1; see also Nokelainen et al. 2012), and Hegna and Mappes (2014), who experimentally tested for a disruptive effect against a green background, found no survival benefit of disruptive versus nondisruptive wing markings when moths were presented on the green background. However, when approached by humans, the moths, especially females, sometimes feign death. In this behavior, they exhibit tonic immobility, adopt a specific posture with folded legs and flattened wings, and drop to the ground (personal observation), where they are very difficult to detect (Fig. 1). Although direct observations in the field are lacking, it is likely that the moths react to approaching birds, their main predators (Nokelainen et al. 2012, 2014), in the same way. These observations of moth behavior led us to hypothesize that disruptive wing patterning might function as cryptic coloration on less contrasting backgrounds (Fig. 1A). If true, then by switching backgrounds (i.e., by dropping to the ground), the moths can instantaneously change the function of their coloration from warning to camouflage. To establish that the disruptive forewing coloration pattern of this moth can have a camouflage effect, we conducted an experiment in which we presented to great tits (*Parus major*) artificial moths with and without a disruptive marginal pattern (based on natural patterns) on a background similar to the natural one and examined their detectability by the birds.

## Materials and Methods

To examine the disruptive function of marginal forewing patterns in *P. plantaginis*, we conducted an experiment in a large aviary by giving bird predators a prey‐searching task. The birds were offered artificial paper moths made with slightly modified natural forewing patterns of *P. plantaginis*. The detectability of marginal (i.e., with brighter color patches extending to the wing margin) and nonmarginal (i.e., brighter color patches did not reach the margin) patterns by the birds was compared to determine whether the marginal patterns had a cryptic advantage. Before the detectability test, we conducted a preference test to see whether the birds showed a significant preference for any particular brightness level or pattern. Because such a preference would bias the detectability test results, we controlled for any bias in the detectability test. The experiment was carried out in March and October 2011 at Konnevesi Research Station in Central Finland.

### Predators

Great tits (*Parus major*) were used as predators in the experiment because they are common visual predators of insect prey in Central Finland. Great tits are also conveniently small (12.5–14.0 cm in length), and they are explorative in captivity, which makes them easy to train for prey‐handling tasks. Using traps, 63 birds were caught at a feeding site and brought indoors. They were housed individually in plywood cages (64 × 46 × 77 cm high) that were illuminated from 08:00 to 18:00 local time. Food (sunflower seeds, peanuts, and tallow) and fresh water were offered ad libitum. After the experiment, the birds were ringed for identification and then released at the site where they had been caught. The experiments, preference test and detectability test, were conducted from 23rd November to 15th December 2011 and from 16th to 31st March 2011, respectively. Permits to keep wild great tits in captivity and use them in research were issued by the Central Finland Regional Environment Center (KESELY/1017/07.01/2010) and the national Animal Experiment Board (ESAVI‐2010‐087517Ym‐23).

### Prey

The artificial prey were paper moths with a piece of almond glued underneath as a reward for the birds. The printed wing patterns were made from modified photographs of *P. plantaginis* forewings. In this species, the coloration of both the fore‐ and hindwings varies on a broad geographic scale (Nokelainen et al. 2012; Hegna et al. 2013), but only forewings were used to craft the artificial prey because the moths often rest in a posture with their forewings covering most of the hindwings (Fig. 1A). The forewing pattern of *P. plantaginis* consists mainly of white‐to‐yellow patches on a black background. From the natural geographic variation of the species, we chose two extreme types, called “Regular” and “Hash”, for the experiment. The Regular pattern has more and slightly larger white‐to‐yellow patches (covering 32.2% of the area of the forewing) than the Hash pattern (19.3%), and all of the Regular pattern patches extend to the edge of the wing. Hash, the more melanistic of the two patterns, has a pair of bars and a spot on each wing, none of which touch the edge of the wing (Fig. 1C). Therefore, Regular, but not Hash, is a potentially disruptive marginal pattern as defined by Stevens and Merilaita (2009b).

The right forewing of a moth with a representative pattern of each type was photographed with a digital camera (FujiFilm Finepix S3 Pro UVIR, Tokyo, Japan) under a light source emitting both visible and UV wavelengths (Arcadia Reptile D3, Salfords, UK). Because we could not use natural light in the indoor experimental arena, we converted the chromatic information of the captured image (Fig. 1C) to an achromatic scale to avoid any bias caused by the unnatural light source. Also, there is some evidence that early object recognition (Pearson and Kingdom 2002) is more strongly influenced by achromatic (luminance) than by chromatic (color) contrasts in an avian forager (Jones and Osorio 2004). Using Adobe Photoshop software (Adobe Systems Inc., San Jose, CA), we first converted the chromatic color values of each pixel of the image to gray scale (range: 0–255), keeping the natural achromatic contrast between the pale patches and dark coloration of the wing. We, then, picked up average gray tones (pale: 236 and dark: 34) from the image and replaced all pale patches and dark coloration to them. A mirror image of the right forewing image was then made and used for the left forewing to produce a symmetric artificial moth. The moth images were printed with an HP Color Laserjet CP2025 printer on all‐weather copier/laser paper (Rite in the Rain©; J. L. Darling Corporation, Tacoma, WA), so that the printed images were approximately the same size as actual *P. plantaginis* specimens (2 cm wingspan). The reflectance of the printed images and the experimental background (see below) was also measured with a spectrophotometer (Maya 2000 pro, with a PX‐s pulsed xenon light source, Ocean Optics, Dunedin, FL) to examine coloration of the printed images in relation to avian vision (Fig. S1). To make the artificial moths attractive to the birds, we used nontoxic glue (UHU Stick) to attach a piece of almond underneath the paper wings as a reward. Unlike real *P. plantaginis*, the prey items offered to the birds had no secondary defenses because our focus was on the cryptic function of the forewing pattern.

**Figure 2:**
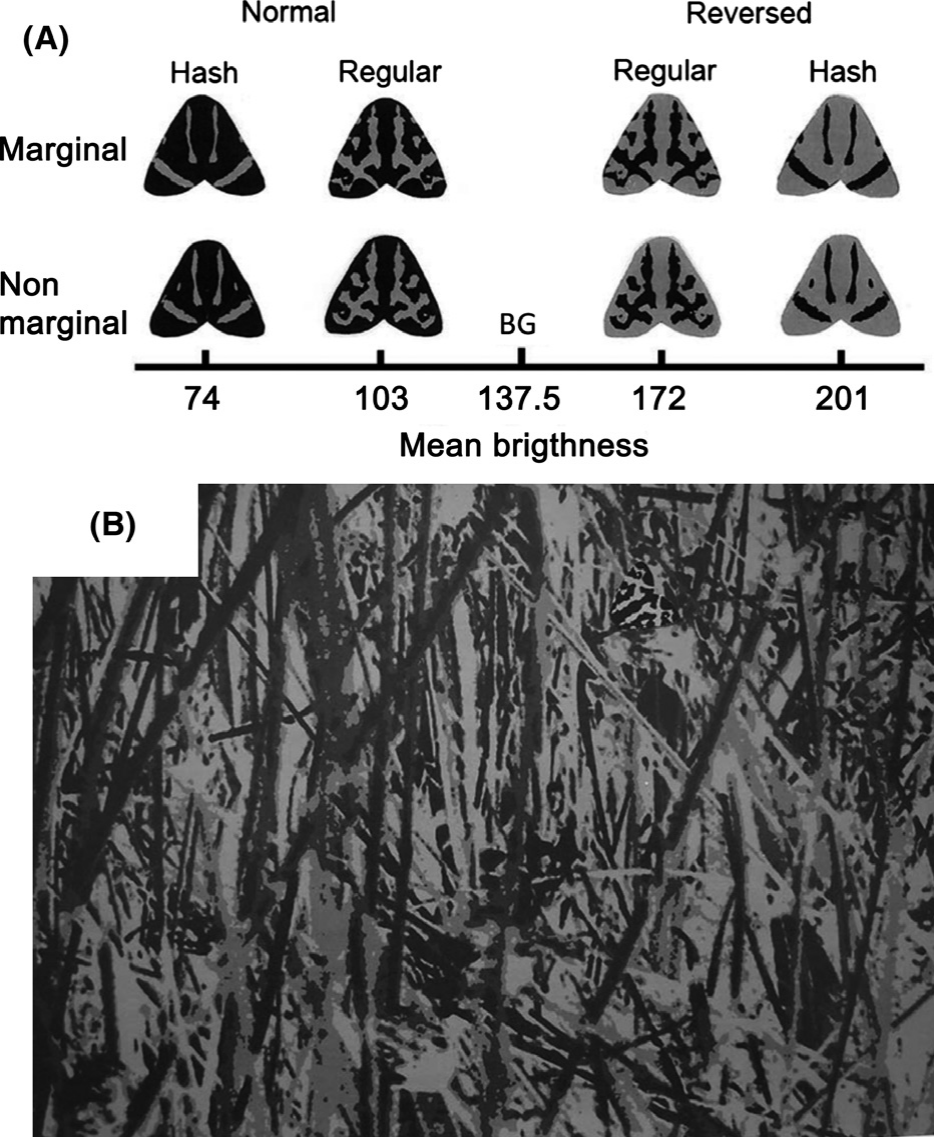
(A) Mean brightness values of gray tone pixels (range, 0–255) of artificial prey and the background (BG) used in the detectability experiment. The eight prey types are Normal marginal Hash, Normal nonmarginal Hash, Normal marginal Regular, Normal nonmarginal Regular, Reversed marginal Regular, Reversed nonmarginal Regular, Reversed marginal Hash, and Reversed nonmarginal Hash. The prey items were created from two natural forewing patterns of *P. plantaginis* (see Materials and Methods). The brightness of the pale and dark gray patches was 236 and 34, respectively. (B) The background used in the detectability test. A Normal marginal Regular prey item is located near the top and slightly to the right of the centerline.

To examine whether the marginality of the patches itself increased concealment, we modified the positions of the pale patches of both wing pattern types while controlling for five possible confounding effects as follows: (1) We kept the total patch area and the general shape of the patches constant; (2) We balanced the direction of pattern manipulation by changing marginal patterns to nonmarginal and vice versa; (3) We counterbalanced the brightness of the prey by reversing the pale and dark areas of each pattern type; (4) We crafted the background such that none of the grays of the background elements exactly matched the grays of any of the pattern elements of the prey. This allowed us to control for the background‐matching effect of the patterned prey while simultaneously matching the brightness of all prey types to the background to the same degree. (5) We tested whether the bird predators showed any preference for a specific brightness level or pattern. We constructed eight prey types divided into two groups of four (normal and reversed); each group of four prey items comprised two pairs, a Hash pair and a Regular pair, and each pair comprised a moth with a marginal pattern and another with a nonmarginal pattern (Fig. 2A). Below, we explain how these procedures were carried out.

(1) We made the counterpart prey items (i.e., moths with a nonmarginal Regular pattern and a marginal Hash pattern) by slightly modifying the position of the pale patches on the wings while keeping their total area constant; thus, the pattern size and brightness were unchanged. (2) We modified the Regular pattern by moving the pixels of each patch that touched the wing edge inward while keeping the overall configuration of the pattern unchanged. For the Hash prey, we did the opposite manipulation; we extended the pale bars in the middle of the forewings so that their ends reached the wing edge by moving edge pixels to the tip of each bar. We also moved the small spots to the nearest wing edge (Fig. 2A). We hypothesized that the modification of the Regular wing pattern would decrease the survival rate of the prey by eliminating the marginality of the pattern. We also hypothesized that the modification of the Hash wing pattern would not affect the detectability of the prey items because the total area of the patches touching the edge was small and, thus, would be unlikely to have any disruptive effect. We included the Hash pattern, both original and manipulated, to test whether the modification of the wing pattern per se would affect prey detectability by the predator. Thus, the four treatments of the normal group were marginal Regular, nonmarginal Regular, nonmarginal Hash, and marginal Hash.

(3) Because the total area of the pale patches differed between Regular and Hash prey, the brightness of each type also differed. Therefore, we crafted reversed patterns for each prey type by inverting all of the achromatic color components of the wings (Fig. 2A). As a result, the brightness of the Hash treatments became higher than that of the Regular treatments but the intensity of the contrast between pattern elements was unchanged. The four treatments of the reversed group were Reversed marginal Regular, Reversed nonmarginal Regular, Reversed nonmarginal Hash, and Reversed marginal Hash (Fig. 2A).

### Experimental background

(4) The prey items were presented to the bird predator on a printed background that we made by manipulating a photograph of the moth's natural habitat in gray scale (Fig. 2B). Because we were interested in the disruptive effect of the marginal wing pattern of *P. plantaginis*, we constructed the background to isolate that effect and to eliminate any possibility of background matching. Natural color patterns of wild animals often comprise both disruptive and background‐matching elements that hinder their detection or recognition, a phenomenon known as “differential blending” (Stevens and Merilaita 2009b; see also Thayer 1909; Cott 1940). To produce the background, we photographed a typical habitat of *P. plantaginis* with a digital camera (DiMAGE X31; Konica Minolta, Tokyo, Japan). Then, using Adobe Photoshop software (Adobe Systems Inc.), we adjusted the size of the image so that the relative size of the artificial prey compared to the background elements was natural. We next replaced the colored elements in the photograph with four shades of gray, none of which were exactly matched the gray shades used for the moth prey as follows: After converting the image into gray scale (255 achromatic color tones), we used ImageJ software (Rasband 1997–2012; Abràmoff et al. 2004) to reduce the 255 tones to four, each including approximately 25% of the total pixels. The four gray tones were produced as follows: Using Photoshop, we counted the number of pixels in the pale patches and the number in the dark base of each marginal and nonmarginal pair of the Normal and Reversed groups (i.e., Normal Hash, Normal Regular, Reversed Regular, and Reversed Hash pairs). Then, we calculated the mean brightness of the pixels of each pair (74, 103, 172, and 201 in gray color tone) and used the mean brightness of each pair as one of the four gray tones of the final background image. We used four copies of the gray‐scale image to make the background. On the first image, we manually chose a gray tone value so that 25% of the total pixels had an equal or lower tone level. Then, we replaced the tones of the selected pixels with the darkest gray of the four tones and made the remaining 75% of the pixels transparent. In the same way, we replaced the tones of 50% and 75% of the pixels on the next two images with the third and second darkest grays, respectively. Finally, we replaced all of the pixels of the fourth image with the palest gray shade. To produce the final background image, we stacked the images with the fourth, palest image on the bottom, overlaid by the “75%,” “50%,” and “25%” images, in that order. As a result, the number of pixels with each of the four gray tones was approximately equal in the final background image (Fig. 2B).

The background images were printed with an HP Color Laserjet CP2025 printer on A4‐sized Rite in the Rain© all‐weather copier/laser paper (J. L. Darling Corporation). The paper backgrounds were then glued onto 3‐mm‐thick A4‐sized plastic corrugated boards to make them more durable and easy to handle. To explore color contrasts between the gray tones of the prey and the experimental background for the bird vision, we calculated achromatic color distances (Vorobyev & Osorio 1998) between used gray tones (of the prey and the experimental background) under the illumination in aviary where the experiment was conducted. As vision system of the great tit is not well known, we calculate color distances with vision system of the closely related blue tit (*Cyanistes caeruleus*) (Hart et al. 2000). Avian vision model analysis was conducted with R 3.0.2 and Pavo package (Maia et al. 2013). The light gray tone of the prey appeared to deviate from all the four shades of the experimental background (6.8, 1.7, 4.2, and 9.9) more than one unit of Just Noticeable Difference (JND), which is considered to be a threshold for two colors to be discernible for a receiver (Vorobyev & Osorio 1998). Darker gray tone of the prey, however, deviated more than one JND from all except darkest tone of the experimental background (0.4, 5.5, 11.4, and 17.2).

### Bird training

Prior to the experiments, all predators (34 great tits for the detectability test, and 29 different great tits for the preference test) were trained to feed on artificial moths placed on an artificial background in the cages in which they were housed. The birds learned to associate the artificial paper prey with the almond reward. The training prey items were the same size and shape as the experimental prey, but the printed pattern was fine pale gray dots. The experimental background was used to train the birds for the detectability test, and the background used to train birds for the preference test consisted of a piece of A4‐sized paper divided into four rectangular sectors, two black and two white, arranged in a checkerboard pattern.

### Preference test

(5) The preference test was conducted to determine whether the birds showed a significant preference for any particular prey type, which would bias the results of the detectability experiment. In the preference test, all eight types of prey were highly visible to the birds and presented simultaneously. The 29 birds used in the preference test were not used in the detectability test. Each bird was introduced into a plywood experiment cage (50 × 70 × 70 cm high) containing only a perch and a water bowl. The cage was illuminated with a full‐spectrum terrarium lamp (Repti Glo compact 2.0; EXO TERRA; Rolf C. Hagen Inc., Montreal, Canada). Because the brightness of the four non‐marginal–marginal prey pairs differed, we controlled for a possible interaction between the brightness of the prey and that of the background using three artificial backgrounds: white, black, and checkered (with 1.5 × 1.5 cm white and black squares). In the experiment, each bird was presented the prey items on all three backgrounds, one background at a time, and the order of presentation was balanced among the birds. We punctured 12 holes (about 7 mm diameter) in equally distributed grid (distance between preys about 5 cm) in each background and attached one of each of the eight prey types in random position; a small piece of almond underneath of each prey item was housed within the hole such that the prey item would not be “bulge” on the background sheet; the undersurface of the prey items were attached with a small piece of cellophane tape (about 2 × 3 mm) so that it could not be blown off the sheet by the flying bird. The birds were allowed to attack the prey items and the order in which the different prey types were attacked was recorded. The position of each prey type on the background was also balanced among trials to avoid a possible bias caused by the position itself. For example, prey that were closer to the perch seemed to be attacked sooner.

### Detectability test

The detectability test was conducted in an aviary room with an area of 57 m^2^. We set 240 A4‐sized experimental background sheets on the floor as habitat patches. Half of the patches were randomly assigned one of the 120 prey items (10 replicates of each of the eight prey types and four plain prey, one of each shade of gray used to make the background). We punctured a small hole in a random position in each of the 120 background sheets to which a prey item had been assigned and attached a paper moth in the same way as Preference test.

In each trial (*n *=* *34), a trained bird was released into the arena and allowed to attack (i.e., rip up the paper prey and eat the almond) up to 50 prey items. We observed the foraging behavior from outside of the aviary through a one‐way glass window and recorded the time of each attack from the start of the trial. If a bird did not attack 50 moths, the trial was terminated 60 min after the start. Two water bowls were provided at the corners of the arena, and the birds were allowed to drink ad libitum during the experiment. Each bird was tested only once.

### Data analysis

#### Preference test

The order in which prey were attacked was recorded, and the data were analyzed by a mixed‐effect Cox model by applying the “coxme” function in the R software “coxme” package (v. 2.12.2) (R Development Core Team 2011. In this analysis, “attack order” was the response variable, and background type (white, black, or checkered), mean brightness of the prey of marginal and nonmarginal pairs (Normal Hash* *=* *29, Normal Regular* *=* *40, Reversed Regular* *=* *67, and Reversed Hash* *=* *79), and prey type (Regular or Hash) were fixed explanatory factors. Bird ID was included in the model as a random factor. We also entered third‐order interaction terms of the three explanatory factors to construct the full model. The model with the smallest Akaike information criterion (AIC) value was selected using a backward stepwise procedure.

We also tested whether the position of the patches per se affected the order in which the different prey were attacked. For that purpose, we fitted a mixed‐effect Cox model in which the marginality of the patch (marginal or nonmarginal) and its interaction with prey type (Normal Regular, Reversed Regular, Normal Hash, and Reversed Hash) were included as explanatory factors.

#### Detectability test

Because the preference test results clearly indicated a significant interaction between prey type and brightness of prey (see Results), the data for each of the four prey pairs (Normal Hash, Normal Regular, Reversed Regular, and Reversed Hash) were analyzed separately. The difference in attack risk between the two patch marginality patterns (marginal or non‐marginal) was examined using a mixed‐effect Cox model by applying the “coxme” function in the “coxme” package for R (version 2.12.2; R version 2.11.1 2011). In this analysis, “time to attack” was the response variable, and patch marginality (marginal or nonmarginal) was included as a fixed explanatory factor; bird ID was entered as a random factor.

## Results

### Preference test

**Figure 3:**
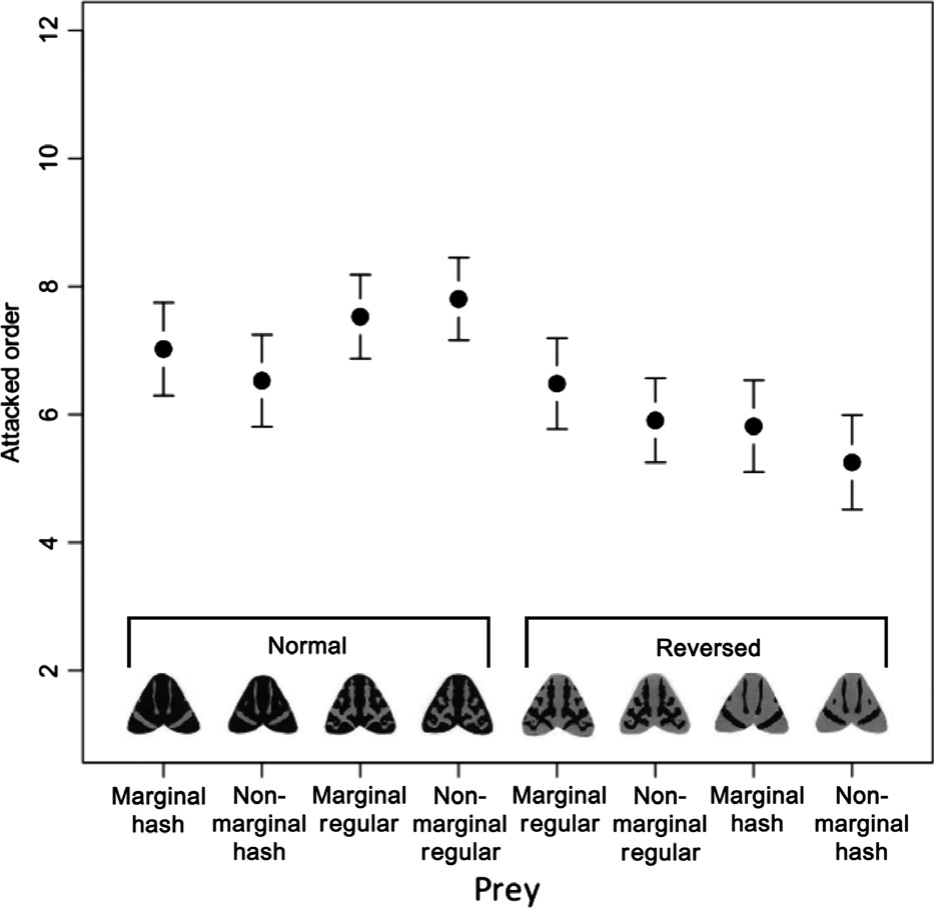
The order in which prey were attacked (mean and 95% CI) in the preference test. The prey items are arranged from left to right according to their brightness value. marginal and nonmarginal members of each pair have the same brightness.

The preference test results showed that the birds preferred certain prey types, although the direction of the bias was not straightforward. The best‐fitting model included four terms: background, prey brightness, prey type, and the interaction between prey type and prey brightness (Table 1; Fig. 3). This result indicated that brightness, which differed among the four prey pairs (see Fig. 2) affected the attack rate, but that the effect was different among the prey types, suggesting that the detectability should be compared within each prey pair. The background type (white, black, or checkered) also affected the attack rate; prey items presented on white backgrounds were attacked earlier, irrespective of prey type and brightness.

**Table 1:** Best‐fitting models obtained by Cox mixed‐effect regression analysis of the preference test results. Bird ID was included as random effect. The asterisk indicates interaction between two variables

Term	coef	se (coef)	*z*	*P*
Background (black vs. checkered)	0.136	0.0931	1.46	0.14
Background (black vs. white)	0.209	0.0933	2.24	0.025
Prey type (Hash vs. Regular)	−0.905	0.294	−3.08	0.0021
Brightness	0.117	0.0359	3.27	0.0011
Prey type*Brightness	0.285	0.113	2.51	0.012

There was no difference in the order of attack between marginal and nonmarginal prey items (Cox regression; exp (coef)* *=* *0.897, *z *= −1.01, *P *=* *0.310), indicating that the birds did not show a preference for wing pattern marginality per se. Nor did the interaction between the position of the patch (marginal or nonmarginal) and prey type (Hash or Regular) significantly affect the attack order (Cox regression; exp (coef)* *=* *1.043, *z *=* *0.27, *P *=* *0.780).

### Detectability test

**Figure 4:**
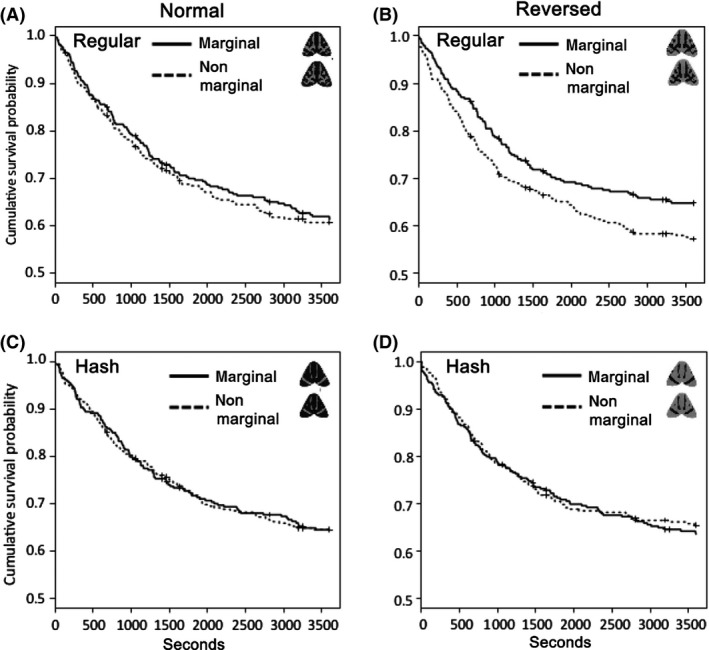
Survival curves of each pair of prey items along the time (sec) from the start of the trial in the detectability test: (A) Normal Regular, (B) Reversed Regular, (C) Normal Hash, and (D) Reversed Hash. The four pairs were analyzed separately because the preference test showed a significant effect of the interaction between prey type and mean prey brightness.

Analysis of the Normal Regular pair showed that the attack risk for prey with a nonmarginal pattern (i.e., nonmarginal Regular) was not significantly higher than that for the original marginal Regular (Cox regression analysis; *n *=* *29, exp (coef) = 1.055, *z *=* *0.43, *P *=* *0.67; Fig. 4A). In the analysis of the Reversed Regular group, the pattern manipulation (from marginal to nonmarginal) significantly increased the risk of attack (Cox regression analysis: Reversed marginal Regular vs. Reversed nonmarginal Regular; exp (coef) = 1.302, *z *=* *2.09, *P *=* *0.036; Fig. 4B); this result supports the hypothesis that a marginal pattern of the wing markings has a disruptive effect in *P. plantaginis*. In contrast, in the analysis of the two Hash pairs (Normal and Reversed), extension of the wing pattern to the wing edge did not significantly affect the attack risk for the prey items. (Cox regression analysis: nonmarginal Hash vs. marginal Hash, exp (coef) = 0.991, *z *= −0.07, *P *=* *0.95; Reversed nonmarginal Hash vs. Reversed marginal Hash, exp (coef) = 1.056, *z *=* *0.13, *P *=* *0.68; Fig. 4C, D).

## Discussion

The results of the detectability test suggest that in *P. plantaginis*, an aposematic moth, a marginal forewing pattern may have a disruptive function but only under certain conditions. We hypothesized that marginal prey items would survive longer than their nonmarginal counterparts in Regular pairs but not in the Hash pairs. The data partly support this hypothesis: marginal markings decreased detectability of the prey in the Reversed Regular pair but not in the Normal Regular pair (Fig. 4). It is not clear why a concealing effect of the marginal patterns was detected only in the Reversed Regular pair and not in the Normal Regular pair. It is possible that the relative efficacy of marginal patterns with respect to background matching differed between prey items that were lighter or darker overall compared to the experimental background. Although we did use the same gray tones in both the Normal and Reversed groups, the prey items in the Reversed pairs might be easier for the birds to detect against the experimental background because reversed prey showed smaller proportion of coloration that could not been discern from one of four background colors by birds (JND 0.4) in our experimental conditions. In this regard, it is noteworthy that, in the Normal Regular pair, the survival curve of marginal Regular prey item, with marginal markings, was always higher than their nonmarginal counterparts, even though we did not find a significant difference in its probability of survival (Fig. 4A). These results seems to concord with Troscianko et al. (2013), in which they generated pairs of marginal and nonmarginal artificial moth with different intra‐object contrasts on the image of natural tree trunk displayed on a computer screen and compared their detectability for human “predators”; the results showed that deference in detection time between marginal and nonmarginal prey was consistently larger in high intra‐object contrasting (and naturally more contrasting surrounding background) pair than lower contrasting one (Troscianko et al. 2013; Fig. 3). In contrast, in both the Normal and Reversed Hash pairs, there seemed to be no consistent difference between the survival curves of the marginal and nonmarginal prey items (Fig. 4C, D).

As we hypothesized, in the Hash pairs (both Regular and Reversed), modifying the wing pattern had no effect on survival. Even though the markings created some false edges, the number of markings was not enough to completely break up the outline of the forewing shape (Fig. 2). This result is also in accordance with that reported by Bohlin et al. (2012), who manipulated images of a firebug so that all of the black elements of the typical warning color pattern were marginal. They found that the rearrangement did not increase the time to detection by human predators. Although the Hash forewing pattern does not provide the moth a disruptive benefit, it may reflect a trade‐off between various possible functions of the wing coloration. For example, although *P. plantaginis* individuals with more melanized hindwings may experience higher attack rates because of a weak warning signal and the lack of a disruptive effect, they may have an advantage with respect to thermoregulation (Hegna et al. 2013). Because the melanization level of the fore‐ and hindwings is related to the geographic distribution pattern of the moth (Hegna and Mappes 2014), it is likely that there is a stronger directional selection for melanization in areas where the Hash pattern is common.

The results of the preference test, which was designed to identify possible biases in the birds, showed that the birds tended to prefer brighter prey items, and the trend was most apparent in the Reversed group (Fig. 3). Sandre et al. (2010) reported that a higher luminance contrast between the color elements of prey, and a higher contrast between pattern elements and the background, led to more frequent attacks by naïve predators. The bias detected in our preference experiment relates to the brightness of the prey pairs; because we used both white and black backgrounds and found no effect of background type on attack order, the mechanism responsible for bias is probably not the same as that suggested by Sandre et al. (2010). It is noteworthy that, in the present study, the birds were trained with palatable artificial prey before the preference test. Therefore, it is likely that higher brightness per se attracts attacks from birds (see also Lyytinen et al. 2004).

The benefits of warning coloration against avian predators are expected to increase with increasing conspicuousness owing to initial avoidance (Gamberale‐Stille and Tullberg 1999; Lindström et al. 1999; Marples et al. 2005; Exnerová et al. 2007), faster avoidance learning (Gittleman and Harvey 1980; Sillén‐Tullberg 1985; Riipi et al. 2001), longer avoidance memory (Roper and Redston 1987; Roper 1994), and enhanced ability to discriminate the aposematic species from alternative palatable prey species (Guilford 1986; Gamberale‐Stille 2000). At the same time, increasing conspicuousness carries with it the cost of higher detection risk (Gittleman and Harvey 1980; Riipi et al. 2001; Summers and Clough 2001; Mappes et al. 2014) by predators that are either naïve to the signal (Marples and Mappes 2011) or tolerant of the prey's defense (Endler and Mappes 2004; Valkonen et al. 2012). Moreover, in some cases at least, as the signal becomes more conspicuous, the benefit derived from the conspicuous signaling decelerates while the cost increases or decelerates less sharply (Stevens and Ruxton 2012). In these cases, natural selection may favor warning signals that are not maximally conspicuous (Endler and Mappes 2004; Speed and Ruxton 2007; Ruxton et al. 2009), or that become less conspicuous from a distance, while being highly conspicuous at close range (Edmunds 1974; Papageorgis 1975; Rothschild 1975; Endler 1978; Järvi et al. 1981). The latter idea is supported by the results of experiments using human predators (Tullberg et al. 2005; Bohlin et al. 2008). *Parasemia plantaginis*, however, is highly conspicuous even at long distances when it is resting on green foliage (Fig. 1). Thus, although the warning coloration may confer an advantage on this species by deterring generalist predators, it also increases the risk from predators that are tolerant to the moth's defense (see Nokelainen et al. 2014). Moreover, Hegna and Mappes (2014) reported that disruptive markings on the forewings do not increase the survival of these moths against a green background. The results of the present study suggest that the Regular forewing pattern of *P. plantaginis* may be part of a two‐step defensive tactic against different types of predators. *Parasemia plantaginis* individuals are very conspicuous (with or without their hindwings exposed) on the green foliage on which they typically rest. Once a predator approaches the moth, which means that the warning signal has not prevented an attack, they have three options to protect themselves against the predator: they can flee, display their colorful hindwings, or feign death. We have repeatedly observed the moths to do all three. Under high temperature conditions, males are particularly likely to try to escape, taking off when disturbed. Under low‐temperature conditions, females, especially, commonly drop off of leaves and hide in the litter below (A. Honma, J. Mappes, and J. K. Valkonen, unpubl. data). The latter behavior seems to be a very good strategy because the disruptive effect of their forewing pattern is enhanced against litter, which, according to our results, can indeed decrease the risk of detection (see Fig. 1).

Sherratt et al. (2005) proposed that individuals with disruptive coloration can exploit a greater range of habitats than cryptic individuals without disruptive patterns, because disruptiveness is less background‐dependent than crypsis. Although some studies have reported that animals can control their conspicuousness by choosing among different backgrounds (e.g., Marshall 2000), disruptive coloration can have a concealing effect even against a background color that does not match the color of the prey. Our results suggest that a novel advantage of disruptive coloration may be that it affords prey the opportunity to switch the function of their body markings from a conspicuous warning signal to crypsis by moving to a different background.

## Conflict of Interest

None declared.

## Supporting information


**Figure S1.** Spectral reflectance curves of the gray color tones of the prey and the experimental background. Gray dashed line represents lighter gray tone of the prey, and black dashed line represents darker one of the prey. Solid black lines stand for gray tones of the experimental background.
