# Supplementary figures and images for: Warning coloration can be disruptive: aposematic marginal wing patterning in the wood tiger moth
Source: Ecol Evol. 2015 Oct 12;5(21):4863–74. doi: 10.1002/ece3.1736 (PMC4662304; doi:10.1002/ece3.1736)

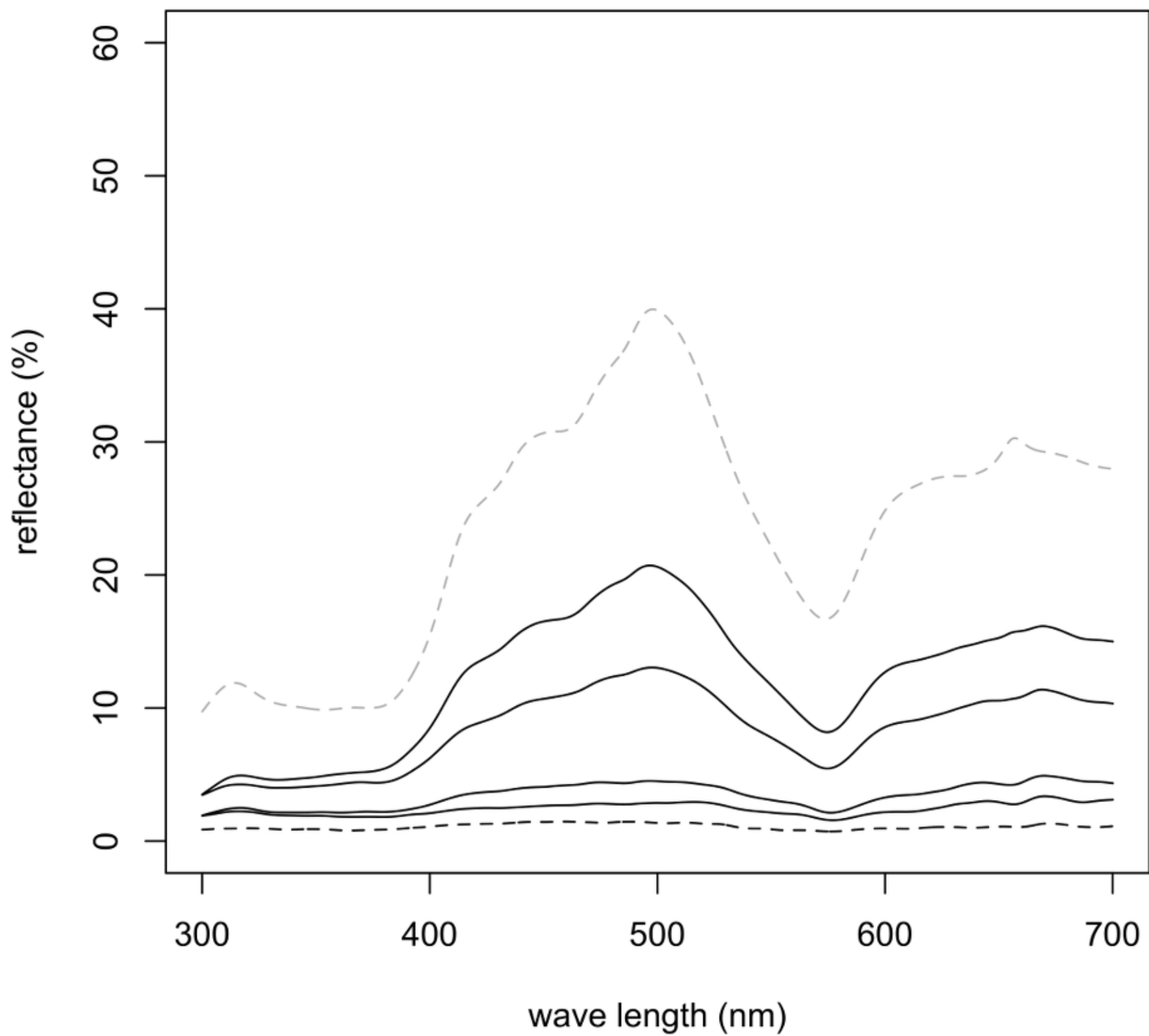

Supplement: Supplementary file 1 — Figure S1. Spectral reflectance curves of the gray color tones of the prey and the experimental background. Gray dashed line represents lighter gray tone of the prey, and black dashed line represents darker one of the prey. Solid black lines stand for gray tones of the experimental background. [file ECE3-5-4863-s001.pdf]
